# Open lift–drill–fill–fix for medial osteochondral lesions of the talus: surgical technique

**DOI:** 10.1007/s00064-023-00833-7

**Published:** 2023-10-12

**Authors:** Quinten G. H. Rikken, Barbara J. C. Favier, Jari Dahmen, Sjoerd A. S. Stufkens, Gino M. M. J. Kerkhoffs

**Affiliations:** Department of Orthopedic Surgery and Sports Medicine, University of Amsterdam, Meibergdreef 9, 1105AZ Amsterdam, The Netherlands

**Keywords:** Osteochondral lesion, Talus, OLT, Fixation, Open, Osteochondrale Läsion, Talus, OLT, Fixierung, Offen

## Abstract

**Objective:**

Osteochondral lesions of the talus (OLT) with a fragment on the talar dome that fail conservative treatment and need surgical treatment can benefit from in situ fixation of the OLT. Advantages of fixation include the preservation of native cartilage, a high quality subchondral bone repair, and the restoration of the joint congruency by immediate fragment stabilization. To improve the chance of successful stabilization, adequate lesion exposure is critical, especially in difficult to reach lesions located on the posteromedial talar dome. In this study we describe the open Lift, Drill, Fill, Fix (LDFF) technique for medial osteochondral lesions of the talus with an osteochondral fragment. As such, the lesion can be seen as an intra-articular non-union that requires debridement, bone-grafting, stabilization, and compression. The LDFF procedure combines these needs with access through a medial distal tibial osteotomy.

**Indications:**

Symptomatic osteochondral lesion of the talus with a fragment (≥ 10 mm diameter and ≥ 3 mm thick as per computed tomography [CT] scan) situated on the medial talar dome which failed 3–6 months conservative treatment.

**Contraindications:**

Systemic disease, including active bacterial arthritis, hemophilic or other diffuse arthropathies, rheumatoid arthritis of the ankle joint, and malignancies. Neuropathic disease. End-stage ankle osteoarthritis or Kellgren and Lawrence score 3 or 4 [3]. Ipsilateral medial malleolus fracture less than 6 months prior. Relative contra-indication: posttraumatic stiffness with range of motion (ROM) < 5°. Children with open physis: do not perform an osteotomy as stabilization of the osteotomy may lead to early closure of the physis, potentially resulting in symptomatic varus angulation of the distal tibia. In these cases only arthrotomy can be considered.

**Surgical technique:**

The OLT is approached through a medial distal tibial osteotomy, for which the screws are predrilled and the osteotomy is made with an oscillating saw and finished with a chisel in order to avoid thermal damage. Hereafter, the joint is inspected and the osteochondral fragment is identified. The cartilage is partially incised at the borders and the fragment is then lifted as a hood of a motor vehicle (lift). The subchondral bone is debrided and thereafter drilled to allow thorough bone marrow stimulation (drill) and filled with autologous cancellous bone graft from either the iliac crest or the distal tibia (fill). The fragment is then fixated (fix) in anatomical position, preferably with two screws to allow additional rotational stability. Finally, the osteotomy is reduced and fixated with two screws.

**Postoperative management:**

Casting includes 5 weeks of short leg cast non-weightbearing and 5 weeks of short leg cast with weightbearing as tolerated. At 10-week follow-up, a CT scan is made to confirm fragment and osteotomy healing, and patients start personalized rehabilitation under the guidance of a physical therapist.

## Abstract (de)

**Operationsziel:**

Osteochondrale Läsionen des Talus (OLTs), im Besonderen mit einem Fragment des Talusdoms, können gut mit einer Fixation in situ behandelt werden. Die Vorteile dieser Behandlung bestehen in der Erhaltung des nativen Knorpels, der Wiederherstellung des subchondralen Knochens wie auch der Wiederherstellung der Gelenkkongruenz durch die direkte und stabile Fixation des Fragmentes. Um eine stabile Fixation zu erreichen, ist es wichtig, die Läsion gut darstellen zu können, insbesondere bei schwierig zugänglichen Läsionen auf dem posteromedialen Dom des Talus. In dieser Studie beschreiben wir die Lift-drill-fill-fix(LDFF)-Methode, eine offene Operationstechnik für osteochondrale Läsionen der medialen Talusrolle mit einem osteochondralen Fragment. Dabei muss die Läsion als eine intraartikuläre Pseudarthrose betrachtet werden, zu deren Behandlung Débridement, Knochentransplantation und stabile Fixation mit Kompression nötig sind. Die LDFF-Technik vereinigt diese Faktoren, wobei ein Zugang mittels einer Osteotomie des medialen Malleolus erreicht wird.

**Indikationen:**

Symptomatische osteochondrale Läsion der medialen Talusrolle mit einem Fragment (≥ 10 mm Durchmesser und ≥ 3 mm Dicke gemäß Computertomographiescan), nach erfolgloser konservativer Behandlungsdauer von 3 bis 6 Monaten.

**Kontraindikationen:**

Systemische Erkrankung einschließlich aktiver bakterieller Arthritis, hämophiler oder anderer diffuser Arthropathien, rheumatoider Arthritis des Sprunggelenks und Malignität. Neuropathische Erkrankung. Arthrose im Endstadium oder Kellgren- und Lawrence-Score 3 oder 4. Fraktur des ipsilateralen Innenknöchels vor weniger als 6 Monaten. Relative Kontraindikation: Arthrofibrose mit ROM („range of motion“) < 5 Grad.

**Operationstechnik:**

Der Zugang zum OLT erfolgt über eine mediale distale Tibiaosteotomie des medialen Malleolus, wobei die Schrauben für die Fixation der Osteotomie vor der Osteotomie gebohrt werden. Die Osteotomie wird mit der oszillierenden Säge ausgeführt, die Vollendung der Osteotomie erfolgt mit einem Meißel, um thermischen Schaden am Knorpel zu vermeiden. Danach erfolgt die Inspektion des Gelenkes, und das Osteochondralfragment wird identifiziert. Der Knorpel wird teilweise entlang des Fragmentrandes mit dem Messer inzidiert und das Fragment schließlich wie eine Motorhaube aufgeklappt. Danach Débridement des subchondralen Knochens, Anbohren des Defektes zur Knochenmarkstimulation (Drill), wonach der Defekt mit autologer Spongiosa, entweder entnommen am Beckenkamm oder der distalen Tibia, aufgefüllt wird (Fill). Schließlich wird das Fragment in anatomischer Position mit einem geeigneten Implantat fixiert (Fix), bevorzugt mit 2 Schrauben, um eine rotationsstabile Fixation zu erreichen. Die Osteotomie des Malleolus wird ebenfalls mit 2 Kortikalisschrauben fixiert.

**Weiterbehandlung:**

Ein Unterschenkelliegegips wird für 5 Wochen angelegt, für weitere 5 Wochen eine Unterschenkelschiene mit schmerzadaptierter Vollbelastung. Danach folgt eine individualisierte Rehabilitationsbehandlung.

## Introductory remarks

Osteochondral lesions of the talus (OLTs) concern lesions of the articular cartilage in combination with the subchondral bone. Patients with symptomatic OLT typically present with deep ankle pain, especially during or after weightbearing, and are not limited to swelling, range of motion restrictions, and locking of the ankle [18]. These complaints can have a significant impact on patients’ ability to participate in sports and quality of life [6]. The first-line treatment for OLTs consists of conservative management; however, in up to 55% of patients, conservative treatment fails and, ultimately, surgical treatment is warranted [20].

When considering surgical treatment for OLTs it is crucial to follow a patient-individualized approach, incorporating patient and (morphological) lesion characteristics in determining the optimal treatment method [18]. In primary, noncystic, lesions up to 10–15 mm in diameter, arthroscopic debridement and bone marrow stimulation is the preferred treatment method [15]. Alternatively, for larger (> 15 mm diameter) and/or cystic lesions, autografting, scaffolding techniques, or allografting are available [13, 18]. In case of an OLT with osteochondral fragment, fixation can be considered [17]. The theoretical benefits of fixation include the preservation of the native cartilage, high quality subchondral bone repair, and restoration of the joint congruency by immediate stabilization of the fragment [5, 14, 16]. Previously, the arthroscopic *Lift, Drill, Fill* and *Fix *(LDFF) technique was described as a promising fixation method and showed excellent results up to long-term follow-up [5, 16, 19]. OLTs that can be treated arthroscopically are usually located anteriorly [8]. However, although lesions can be reached arthroscopically, incomplete access may impede the ability to effectively reduce and fixate the osteochondral fragment with perpendicular screw placement, which can lead to treatment failure [11]. Even more so, lesions located posteriorly are challenging to reach and fixate by means of anterior ankle arthroscopy, and it is known that more than half of OLTs are located on the posteromedial and centromedial zones [2]. In these lesions, an open technique could provide an alternative approach and excellent exposure to the talar dome, which is crucial for effective reduction of the osteochondral fragment and prevention of union complications [5, 12, 21]. To date, however, the open LDFF approach for medially located OLTs has not yet been described and previous studies reporting on other means of open fixation of OLTs have not specifically focused on this surgical technique [7–10, 20]. A clear surgical description for fixation of OLTs will aid surgeons in expanding their treatment options tailored to the individual patient. Thus, the purpose of the present surgical technique paper is to describe the open LDFF surgical technique for symptomatic medial fragmentous osteochondral lesions of the talus.

## Surgical principles and objective

Primary acute and chronic osteochondral lesions of the talus with a fragment, with a minimum diameter of 10 mm and 3 mm depth on computed tomography (CT scan), are suitable candidates for fixation with this technique, as it allows for immediate stabilization of the fragment and restoration of the talar congruency, preservation of the native hyaline cartilage and the initiation of subchondral bone plate healing [5, 17]. The four-step LDFF approach aims to provide both biological healing by the introduction of marrow cells as well as stable biomechanical fixation to allow for optimal union of the fragment and subchondral bone plate healing. In essence, the LDFF can be seen as an intra-articular nonunion repair with debridement and bone grafting, which provides stability and compression. Access to the talar dome can be obtained for lesions located medially through a medial distal tibial osteotomy, which allows for adequate working space and correct screw placement.

### Advantages

- Preservation of the hyaline cartilage
- High-quality subchondral bone repair by bone marrow stimulation and additional cancellous bone grafting
- Excellent exposure
- No harvest site complications with distal tibia grafting, minimal harvest site complications in iliac crest grafting
- Other surgical salvage options remain possible in case of failed fixation

### Disadvantages

- Access through distal tibia osteotomy
- Potential hardware complications which may lead to the need for hardware removal procedure

### Indications

- Symptomatic osteochondral lesion with a fixable fragment situated on the medial talar dome with a minimum size of > 10 mm diameter and 3 mm in depth measured on CT scan [17]
- Contraindications: systemic disease, including active bacterial arthritis, hemophilic or other diffuse arthropathies, rheumatoid arthritis of the ankle joint, and malignancies
- Neuropathic disease
- End-stage ankle osteoarthritis or Kellgren and Lawrence score 3 or 4 [3]
- Ipsilateral medial malleolus fracture less than 6 months prior
- Relative contra-indication: posttraumatic ankle stiffness with ROM < 5°
- Children with open physis: do not perform an osteotomy as stabilization of the osteotomy may lead to early closure of the physis potentially resulting in symptomatic varus angulation of the distal tibia. In these cases only arthrotomy can be considered.

### Patient information

- Surgical risks include infection, hematoma, thromboembolic events, wound healing problems, and transient or permanent nerve damage leading to hypaesthesia of the saphenous nerve.
- Non-weightbearing cast for 5 weeks, followed by a walking boot for another 5 weeks. Hereafter, patient individualized rehabilitation 3–6 months after cast removal guided by a physical therapist
- Late or early screw discomfort requiring removal after consolidation
- Adverse treatment events include fragment delayed—or nonunion, or osteotomy delayed—or nonunion

### Preoperative work-up

- Clinical evaluation, including patient history and physical examination is performed for all patients at the outpatient clinic in order to assess symptoms befitting an OLT. Additionally, care is taken to assess any relevant coexisting pathologies of the foot and ankle which may warrant treatment, such as symptomatic ankle instability which is frequently encountered in patients with OLT [1, 22].
- Radiological assessment of the lesion is preferably carried out through a preoperative computed tomography (CT) scan to assess the three-dimensional lesion and fragment size, lesion location, as well as the lesion and fragment morphology.
- Additionally, the CT scan is used for preoperative planning in order to determine the surgical approach and osteotomy orientation based on the lesion location as well as to assess the need for additional debridement and filling of possible cysts situated below the osteochondral fragment. In case additional cancellous bone is required to fill the lesion site before fixation cancellous bone grafts can be obtained from the distal tibial metaphysis after osteotomy, or the iliac crest as described in a previously published surgical technique [4].
- Lastly, clinical and radiographic work-up by means of weightbearing x‑ray should be conducted in cases of suspected hindfoot malalignment as it may be necessary to address these concomitantly [18].

### Instruments and implants

- Standard orthopaedic set
- Hohman retractors
- Bone rongeur
- Oscillating saw
- Chisel set (including thin blades)
- 2.0 mm Kirschner wires
- 2.0 mm drill
- Coagulation knife
- 3.5 mm cortical screws or a headless alternative
- Large Weber clamps
- Screw or biomaterials for fragment fixation, not limited to, but options including depending on fragment size and surgeon preference:
  - Bio-Compression screw 2.7 mm (Arthrex Inc., Naples, FL, USA) or poly-L-lactide pins (GRAND FIX, Depuy, USA)
  - Autologous bone pegs harvested from the distal tibia
  - (multiple) chondral darts 1.3 mm (Arthrex Inc., USA), to be used only as an antirotational post, a dart will not give sufficient compression in itself.
  - Self-tapping 2.0 or 2.7 mm cortical screw (Johnson & Johnson, USA)

### Anaesthesia and positioning

- General or spinal anaesthesia
- Patients are placed in a supine position with a thigh tourniquet ipsilaterally
- Preoperative antibiotic prophylaxis with 2 g (or adjusted to weight) of cefazolin is administered intravenously

## Surgical technique

(Figs. 1, 2, 3, 4, 5, 6, 7, 8, 9, 10)

**Fig. 1:**
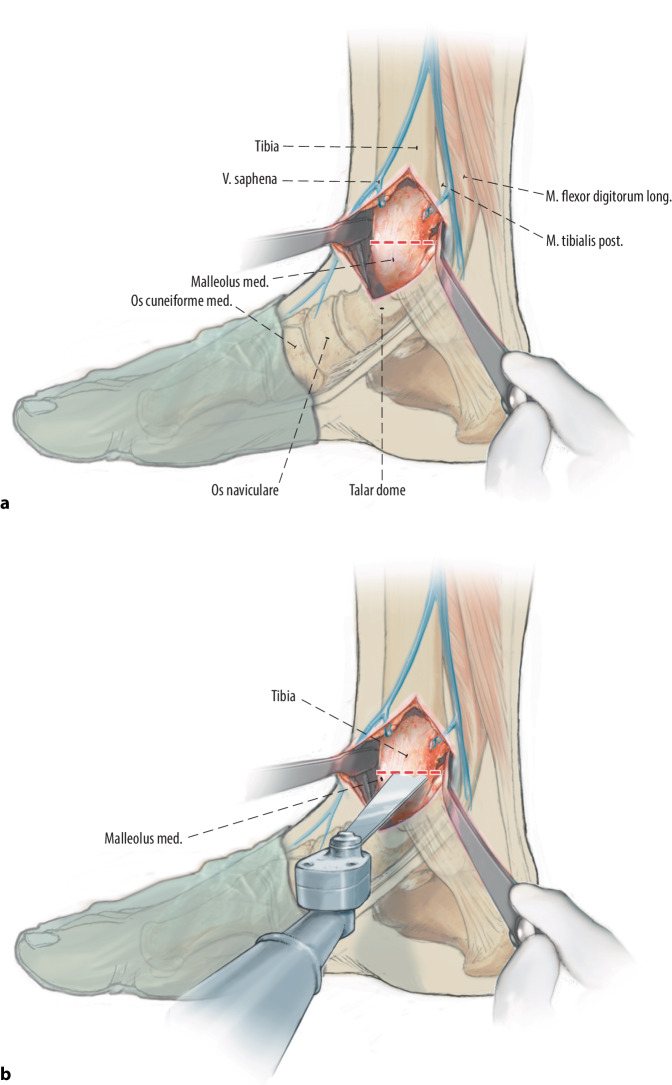
After patient positioning, the work field is carefully prepared by sterile draping of the ankle. The first step is the medial distal tibial osteotomy: an incision is centred over the medial malleolus of approximately 7 cm and lightly curved anteriorly. The large saphenous vein is identified and protected. The arthrotomy is performed anteromedially with a partial resection of the anteromedial joint capsule. In case any anterior distal tibial or anterior medial malleolar osteophytes are present, these are resected using a bone rongeur. Thereafter, the posterior retinaculum of the posterior tibial tendon is released, which is then retracted posteriorly to allow for a limited posteromedial capsule resection. Hohmann retractors are placed both anteriorly and posteriorly of the medial malleolus to protect the tendons and neurovascular structures (**a**). A rolled sterile surgical gown is placed underneath the distal tibia, to allow for the neurovascular structures to move dorsally in order to protect these when performing the osteotomy. Next, when there is good visualization on the extent of the medial distal tibial osteotomy, two cortical lag screw (3.5 mm) holes are predrilled in divergent bicortical manner and measured in anatomical position. Afterwards, the osteotomy is performed with an oscillating saw (**b**). As previously stated, the direction and extent of the osteotomy is determined by the location and size of the osteochondral lesion during preoperative planning. The osteotomy is performed up to around 3 mm from the joint surface and finalized with a broad chisel to avoid (thermal) damage to the surrounding cartilage of the tibial plafond

**Fig. 2:**
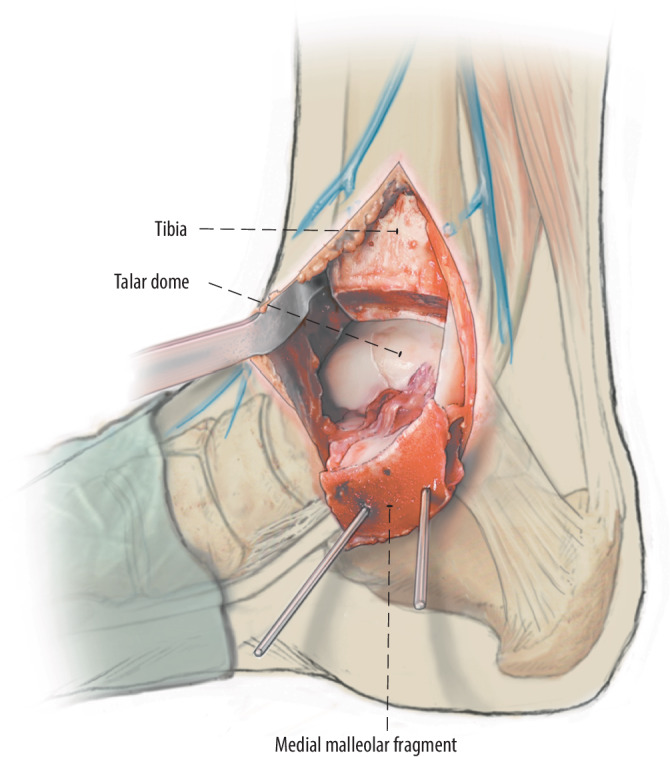
When the osteotomy is completed, the osteotomy site is opened by dislocating the distal tibial fragment of the medial malleolus and fixating it in a plantar and medial direction on the talar body with one or two 2.0 mm Kirschner wires providing stable access to the medial and central talar dome. The Kirschner wires are placed through the drill holes of the osteotomy fixation screws

**Fig. 3:**
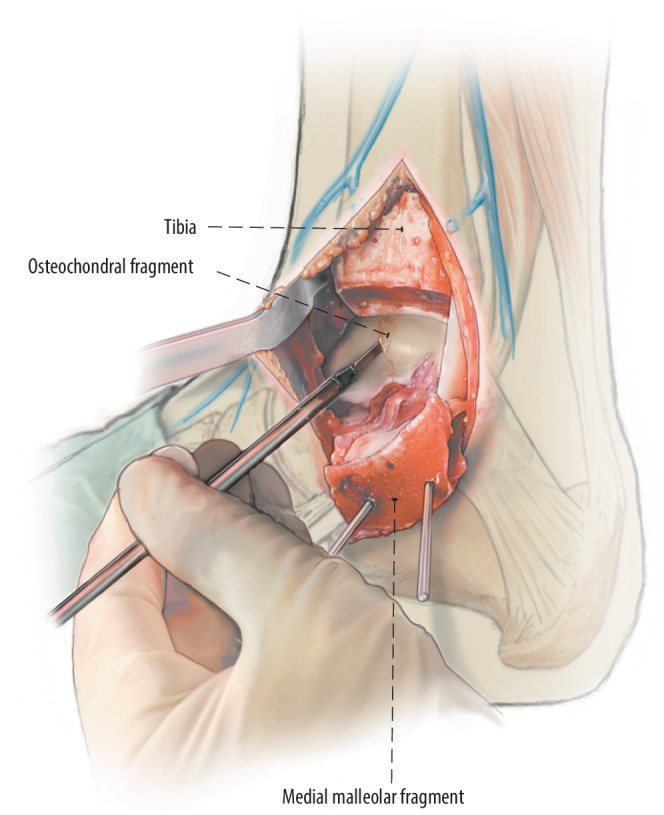
After the osteochondral lesion is identified and inspected, a sharp incision in the cartilage is preferably created with a beaver knife because this allows nice round corners (or any other scalpel to the surgeon’s preference) and a clean lift of the fragment. A semilunar incision is made around the defect, leaving the posterior side of the osteochondral fragment intact if possible. Leaving the posterior side intact provides additional rotational stability and facilitates using only 1 screw in smaller fragments. Of note: in case a fully detached fragment is present, this step is not relevant

**Fig. 4:**
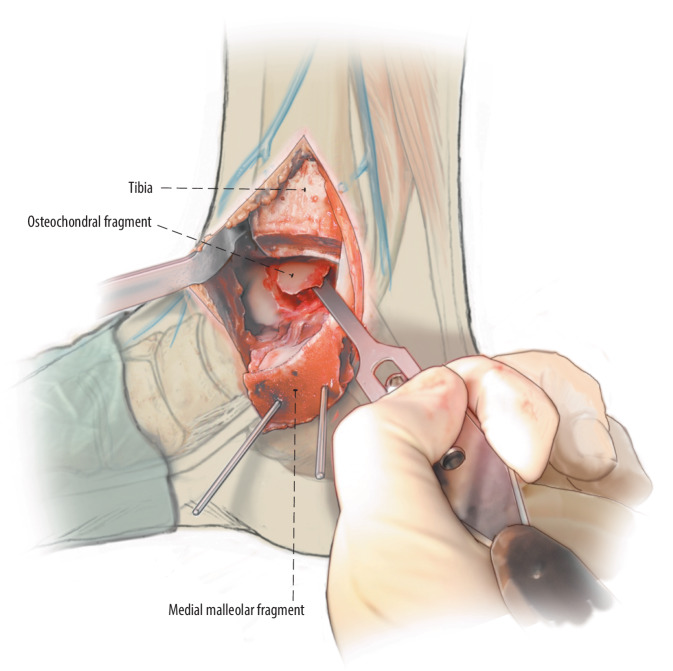
The flap can now be lifted (*lift*) from anteriorly with the use of a chisel. In case the fragment is completely detached in situ before the semilunar incision it is carefully excised and preserved

**Fig. 5:**
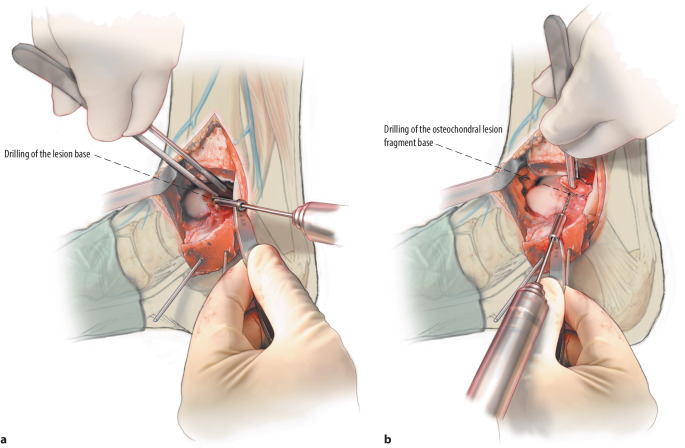
**a**,**b** After lifting or excision of the osteochondral fragment, the subchondral bone is inspected. Promotion of revascularization is key for the surgery to succeed, so all osteosclerotic areas should be diligently debrided, any subchondral cysts should be circumferentially curetted, and the bottom of the surface of the cyst as well as the bony part of the osteochondral talar fragment should be drilled (*drill*) to stimulate underlying bone marrow

**Fig. 6:**
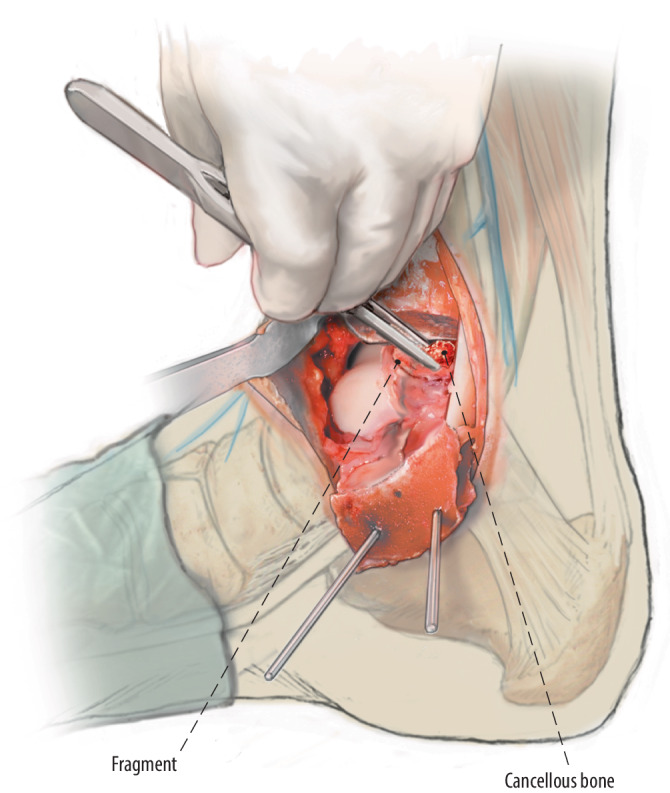
The remaining lesion site after debridement and drilling is filled (*fill*) with autologous cancellous bone harvested from the (exposed) distal tibial metaphysis with a chisel or the iliac crest as described in a previously published surgical technique [4]

**Fig. 7:**
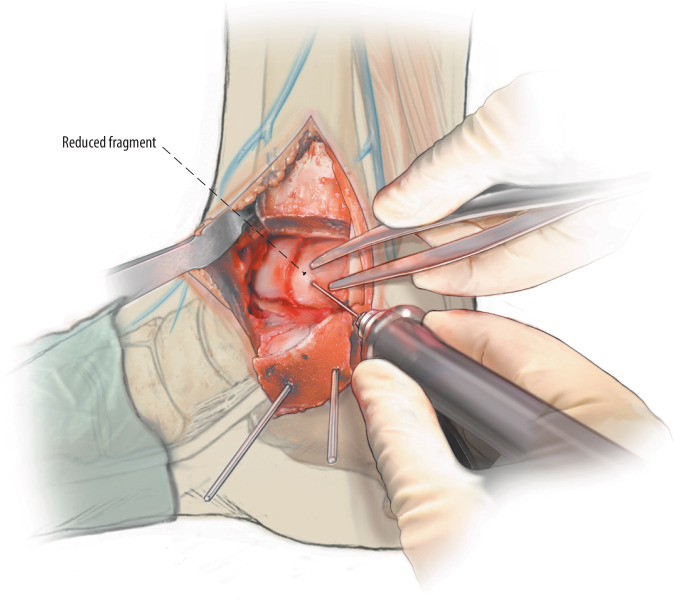
After filling of the defect, the osteochondral flap is reduced to its original position and fixated (*fix*) with a bio-compression screw (Arthrex Inc., USA), poly-L-lactide pins (GRAND FIX, Depuy, USA), or a self-tapping 2.0 or 2.7 mm cortical screw (Johnson & Johnson, USA). Chondral darts (Arthrex Inc.) or bone pegs can be considered in the case of a smaller fragment, or multiple smaller fragments, amendable for fixation

**Fig. 8:**
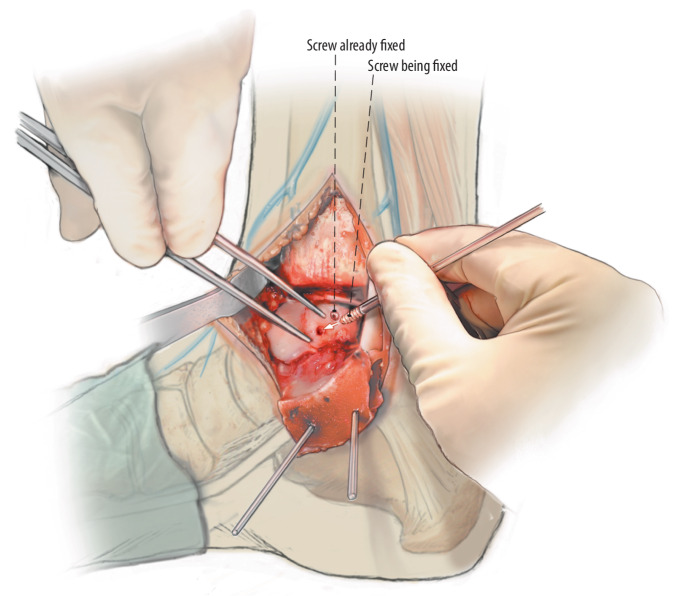
In case of smaller fragments (i.e., < 15 mm diameter), a single screw, centred with adequate compression for good stabilization of the fragment, is placed centrally of the fragment and perpendicular to the lesion site axis. In case of a larger fragment multiple screws can be considered in similar perpendicular orientation to the lesion axis in order to provide rotational stability of the fragment as well

**Fig. 9:**
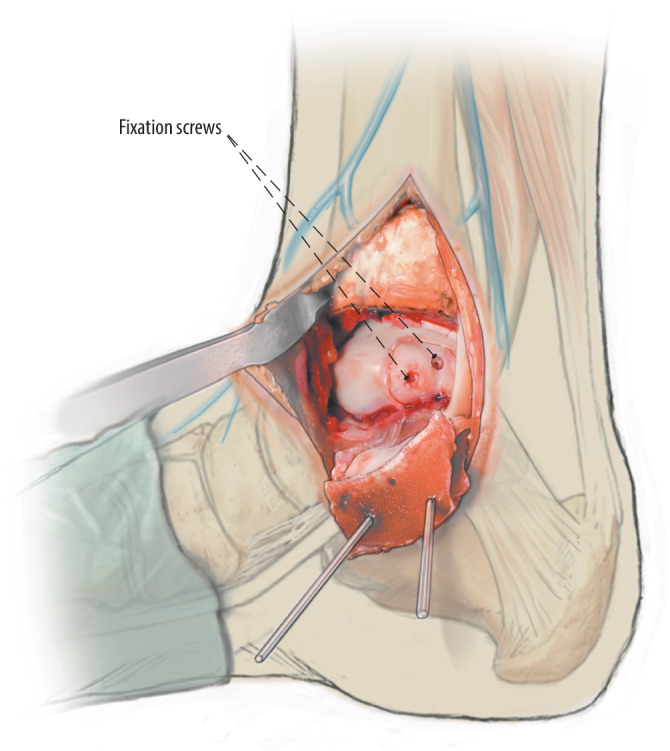
It is important to let the fragment sink discretely (0.5 mm to 1 mm) under talar articular cartilage and to place the fixation screws under this surface to prohibit damage by the screws or darts on the distal tibial articular surface. If a stable fragment reduction and fixation is achieved the joint is carefully inspected and flushed using saline

**Fig. 10:**
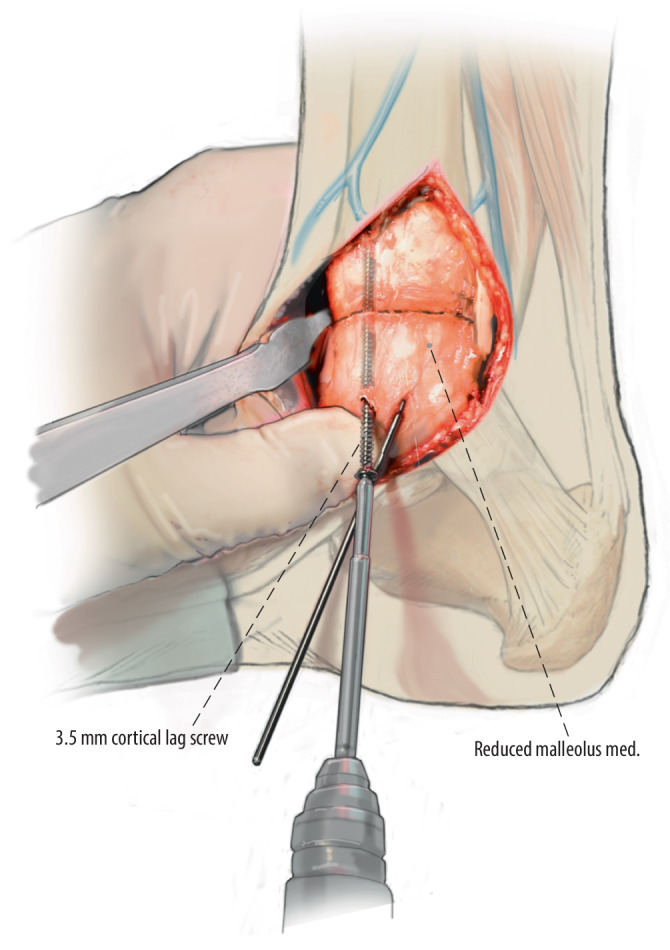
Finally, closure of the osteotomy site is performed. The Kirschner wires are removed, and the medial malleolus is placed in anatomical position and reduced. The two premeasured 3.5 mm cortical lag screws are divergently placed to fixate the osteotomy site and to allow distal tibial bone grafting from the tibial proximal between the two screws. If a large osteotomy was performed, an additional anti-glide plate (1/3 tubular plate) can be added to provide additional rotational and translational stability of the osteotomy. An alternative to the anti-glide plate could be a third cortical lag screw placed transversely in the proximal apex of the osteotomy for additional shear stability. The reduction of the medial distal tibial osteotomy is assessed by means of fluoroscopy

## Special surgical considerations

### Osteotomy.

The authors would like to note that osteotomy choice is surgeon specific and may also include a lateral directed chevron-like osteotomy.

### Grafting.

During the filling of the OLT this surgical technique describes the usage of autologous cancellous bone harvested from the ipsilateral distal tibia osteotomy site or iliac crest in case a larger quantity of cancellous bone is needed to for a large defect site. Graft choice is surgeon specific.

### Postoperative management

**Fig. 11:**
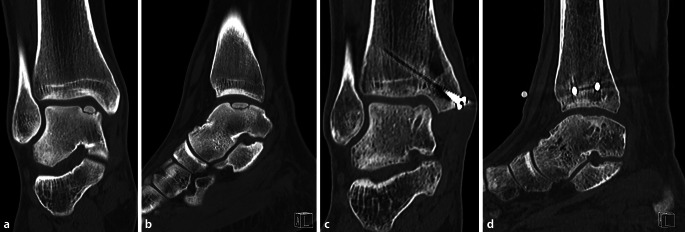
Preoperative (**a**,**b**) and 1‑year postoperative (**c**,**d**) CT scan of LDFF in a large OCL with a fragment. Two bioabsorbable screws were used to achieve a stable fragment fixation as a whole. At 1‑year postoperatively, good union of the fragments and consolidation of the osteotomy had occurred. Of note, the postoperative radiolucency below the fragment is the bioabsorbable screw

- A short leg cast is applied with nonweightbearing for 5 weeks postoperatively and antithrombotic prophylaxis is prescribed for this period. All casts are set in neutral flexion and hindfoot position. One to two weeks postoperatively the non-weightbearing casts consists of a splint to allow for swelling, followed by a circular cast for the remaining time of immobilization. The sutures are removed 2 weeks postoperatively combined with a change of the short leg cast.
- At 5 weeks postoperatively the non-weightbearing cast is exchanged for a short leg walking cast and weightbearing is allowed as tolerated. This cast is applied for 5 weeks.
- Radiographic follow-up with conventional anteroposterior and lateral X‑rays is performed at 5 weeks postoperatively before protected weight-bearing is commenced to reaffirm positioning of the osteotomy. At 10 weeks and 1‑year postoperatively a CT scan is performed in order to assess osteotomy consilidation, fragment consolidation and cyst formation or onset/progression of osteoarthritis (Fig. 11).
- After casting, a patient-centred rehabilitation protocol is started, guided by a physical therapist in order to regain range of motion and muscle strength of the ankle, as well as a normal gait pattern.
- Clinically, the patient is assessed postoperatively. We recommend a follow-up visit at 2, 5, and 10 weeks postoperatively for casting, wound healing, and osteotomy/fragment union consolidation, as well as 6 months and 1 year postoperatively for physical follow-up.

### Errors, hazards, complications

#### Preoperative planning.

- No available CT scan within 1 year of surgery could yield inadequate information regarding the morphology or size of the OLT and osteochondral fragment

#### Surgical technique.

- Inadequate exposure
- No perpendicular screw fixation leading to shallow insertion angle and inadequate compression of the fragment; leading to a higher chance of delayed or nonunion
- Screw size unfit for the fragment size, causing the fragment to break into smaller fragments requiring a salvage procedure (i.e. other OLT surgical treatment) or inadequate compression in larger fragments
- Fragment stabilization or screw too proud (i.e., above the articular cartilage), leading to (early) wear of tibiotalar cartilage

#### Postoperatively.

- Weightbearing too early postoperatively leading to higher risk of osteotomy or fragment nonunion or pseudoarthrosis; possibly requiring revision surgery with a nonunion repair of the osteotomy
- No 10-week postoperative CT scan to assess union of the fragment and osteotomy, which could lead to too early weightbearing and a higher risk of nonunion

#### Postoperative complications.

- Infection, hematoma, thromboembolic events, wound healing problems, and transient or permanent nerve damage leading to hypaesthesia of the saphenous nerve, delayed or nonunion of the osteotomy or fixed fragment.

## Results

This study was approved by the local medical ethics committee of the University of Amsterdam (reference number 08/326). All patients who underwent an open LDFF procedure with a medial distal tibial osteotomy for a symptomatic primary OLT with an osteochondral fragment from January 2017 until January 2021 were prospectively followed up for 2 years. In all, 14 patients with a total of 15 ankles (1 bilateral case) were eligible and included at a mean age of 24 (range 14–46) years. 7 patients were male and 7 were female. Pre- and postoperative outcome assessment was performed with the numeric rating scale (NRS) for pain during rest and walking as well as with the American Orthopaedic Foot and Ankle Society (AOFAS) Ankle–Hindfoot score. Radiological assessment was done with a 3 month CT scan to assess osteotomy union and 1‑year follow-up CT scan to assess fragment union. Lastly, postoperative complications, reoperations, and revision surgeries were assessed.

At final follow-up, 13 out of 14 patients (with 14 out of 15 ankles) were available, and 1 patient was lost to follow-up. The baseline NRS for pain at rest significantly improved from a median 4 out of 10 (interquartile range [IQR] 3–5) to 0 out of 10 (IQR 0–2) at 2‑year follow-up (*P* ≤ 0.05). Moreover, the NRS during walking improved from a baseline median 7 out of 10 (IQR 6–7) to 1 out of 10 (IQR 0–4) (*P* ≤ 0.05). The AOFAS score improved from a median 61 out of 100 (IQR 48–68) at baseline to 95 out of 100 (IQR 76–100) at follow-up (*P* ≤ 0.05). All osteotomies showed union at follow-up CT scans. 14 out of 15 ankles showed union of the osteochondral fragment on 1‑year CT scans. At the 2‑year follow-up, 9 patients had undergone a reoperation, of which 8 patients underwent removal of medial distal tibial screws, with 2 patients additionally undergoing an osteophyte removal and soft-tissue impingement removal, respectively. One patient had a revision procedure by means of a TOPIC autologous bone grafting for recurrent OLT and nonunion of the fragment [4]. Apart from the 1 patient who had nonunion of the osteochondral fragment no postoperative complications were noted.

## Declarations

IRB was obtained before the start of the study. All studies mentioned were in accordance with the ethical standards.
